# Use of retrieval bag in the prevention of wound infection in elective laparoscopic cholecystectomy: is it evidence-based? A meta-analysis

**DOI:** 10.1186/s12893-018-0442-z

**Published:** 2018-11-19

**Authors:** Davide La Regina, Francesco Mongelli, Stefano Cafarotti, Andrea Saporito, Marcello Ceppi, Matteo Di Giuseppe, Antonjacopo Ferrario di Tor Vajana

**Affiliations:** 1 Department of Surgery, Ospedale Regionale di Bellinzona e Valli, Bellinzona, Switzerland; 2 Department of Anaesthesiology, Ospedale Regionale di Bellinzona e Valli, Bellinzona, Switzerland; 3 Unit of Clinical Epidemiology, IRCCS - Ospedale San Martino, Genoa, Italy

**Keywords:** Cholecystectomy, Retrieval bag, Infection

## Abstract

**Background:**

Surgical site infections complicate elective laparoscopic cholecystectomies in 2,4-3,2% of cases. During the operation the gallbladder is commonly extracted with a retrieval bag. We conducted a meta-analysis to clarify whether its use plays a role in preventing infections.

**Methods:**

Inclusion criteria: elective cholecystectomy, details about the gallbladder extraction and data about local or systemic infection rate. Exclusion criteria: cholecystitis, jaundice, concurrent antibiotic therapy, immunosuppression, cancer. A comprehensive literature search of PubMed, Cochrane Library and MEDLINE databases was carried out independently by two researchers, according to the PRISMA guidelines and applying the GRADE approach. Terms used were (“gallbladder”AND(“speciment”OR“extraction”OR“extract”))OR(“gallbladder”OR“cholecystectomy”)AND(“bag”OR“retrieval|”OR|“endobag”OR“endocatch”).

**Results:**

The comprehensive literature revealed 279 articles. The eligible studies were 2 randomized trials and a multicentre prospective study. Wound infections were documented in 14 on 334 (4,2%) patients operated using a retrieval bag versus 16 on 271 (5,9%) patients operated without the use of a retrieval bag. The statistical analysis revealed a risk ratio (RR) of 0.82 (0.41–1.63 95% CI). Concerning sensitivity analysis the estimated pooled RR ranged from 0.72 to 0.96, both not statistically significant. Harbord test did not reveal the occurrence of small-study effect (*p* = 0.892) and the funnel-plot showed no noteworthy pattern.

**Conclusions:**

The results of this review highlight the paucity of well-designed large studies and despite limitations related to the low level of evidence, our meta-analysis showed no significant benefit of retrieval bags in reducing the infection rate after elective laparoscopic cholecystectomy. In absence of acute cholecystitis, accidental intraoperative gallbladder perforation or suspected carcinoma their use, to date, may not be mandatory, so that, further studies focusing on complex cases are needed.

**Electronic supplementary material:**

The online version of this article (10.1186/s12893-018-0442-z) contains supplementary material, which is available to authorized users.

## Background

Laparoscopic cholecystectomy is one of the most common surgical procedures worldwide. Despite being considered a low-risk operation, complications occur and surgical infections have been extensively discussed in literature. The incidence of surgical site infection (SSI) was found to be 2.4–3.2% in a large meta-analysis of studies on perioperative antibiotics in patients undergoing laparoscopic cholecystectomy [1].

In order to avoid surgical site contamination from bile and stone spillage, surgeons pay attention not to open the gallbladder during dissection from the liver bed and retrieval from the abdominal cavity. Depending on the surgeon’s preference, a retrieval bag is used to extract the gallbladder through a trocar incision [2]. Endoscopic bags should be used when gallbladder cancer is suspected, in order to minimize the risk of tumor cell dissemination [3] and in case of acute cholecystitis to avoid spillage of infected bile, stones or pus [4–6]. As a matter of fact, endoscopic bags are commonly used also in elective cholecystectomy despite increased costs and no sound evidence in their favor [7–9]. Are retrieval bags actually useful in preventing wound infections in elective laparoscopic cholecystectomy?

To clarify the question, we conducted a meta-analysis on the available literature.

## Methods

The meta-analysis was performed at San Giovanni Hospital, Bellinzona, Switzerland and was based on previously published studies.

### Search strategy and eligibility criteria

A comprehensive computer literature search of PubMed, Cochrane Library and MEDLINE databases was carried out independently by two researchers, to find relevant published articles (the last search was updated on November 30 2017) according to the Preferred Reporting Items for Systematic Reviews and Meta-Analyses (PRISMA) guidelines [10]. The terms used to search were (“gallbladder”AND(“speciment”OR“extraction”OR“extract”))OR(“gallbladder”OR“cholecystectomy”)AND(“bag”OR“retrieval”OR“endobag”OR“endocatch”). Finally, we searched for additional eligible trials in reference lists of retrieved publications and relevant meta-analyses. No language restrictions were set. The study designs considered eligible for the analysis were prospective and randomized trials. Case reports, retrospective or small case series, letters, editorials, and conference proceedings were excluded.

### Data collection and quality assessment

The following inclusion criteria were applied to select studies for this meta-analysis: elective cholecystectomy, data about the method used to extract gallbladder from the abdomen and data about local or systemic infection rate in the next 30 days after surgery. Exclusion criteria were: acute cholecystitis, jaundice, concurrent antibiotic therapy, immunosuppression, bile or stone spillage, gallbladder cancer.

We extracted study characteristics (author name, publication year, country, sample size, age, study design, inclusion and exclusion criteria, method of randomization, primary and secondary outcomes). Any disagreements between reviewers were resolved by discussion.

Three researchers independently reviewed titles and abstracts of the retrieved articles, applying the above-mentioned selection criteria. The full-text version was then independently evaluated to determine their eligibility for inclusion both in the qualitative and quantitative analysis. The risk of bias and the quality assessments of studies included in the meta-analysis were independently performed by 2 reviewers according to the Study Quality Assessment of Controlled Intervention Studies NHLBI [11]. Ultimately, the overall quality of evidence was graded with Grading of Recommendations, Assessment, Development and Evaluations (GRADE approach) [12]. The quality of evidence was rated with a scale of 4 to 1 (4 = high, 3 = moderate, 2 = low and 1 = very low). Five factors could reduce by 1 or 2 the initial quality of evidence (risk of bias, inconsistency, indirectness, imprecision and publication bias) of selected studies.

### Statistical methods

The power analysis was estimated using 1-sided 2-sample proportion and assuming 5% type I error rate. With a sample size of 334 and 271 for patients operated on in the “with bag” and “without bag” groups respectively, we achieved > 70% power interval. The meta-analysis was performed by pooling the Risk Ratios (RR) of each study, i.e. P1 / P0, where P1 and P0 are the infection rate of patients using or not the retrieval bag. The pooled estimate of RRs was computed by means of the random effects model following the method of DerSimonian and Laird [13]. The Higgins’ I2 index [14] was computed to assess the percentage of total cross-study variation due to heterogeneity rather than chance. Sensitivity analysis was carried out by recalculating the pooled RR after exclusion of each study at a time, to assess the contribution of the study to the RR pooled estimate. For small-study effect was meant that the chance of a smaller study being published was increased if it showed a stronger effect. The small-study effect was assessed by means of the Harbord’s test [15]. The funnel plot was carried out to assess the occurrence of publication bias. STATA software was used for all statistical analyses. (StataCorp. (2015) Stata Statistical Software: Release 14. College Station, TX: StataCorp LP).

## Results

### Literature search

**Fig. 1:**
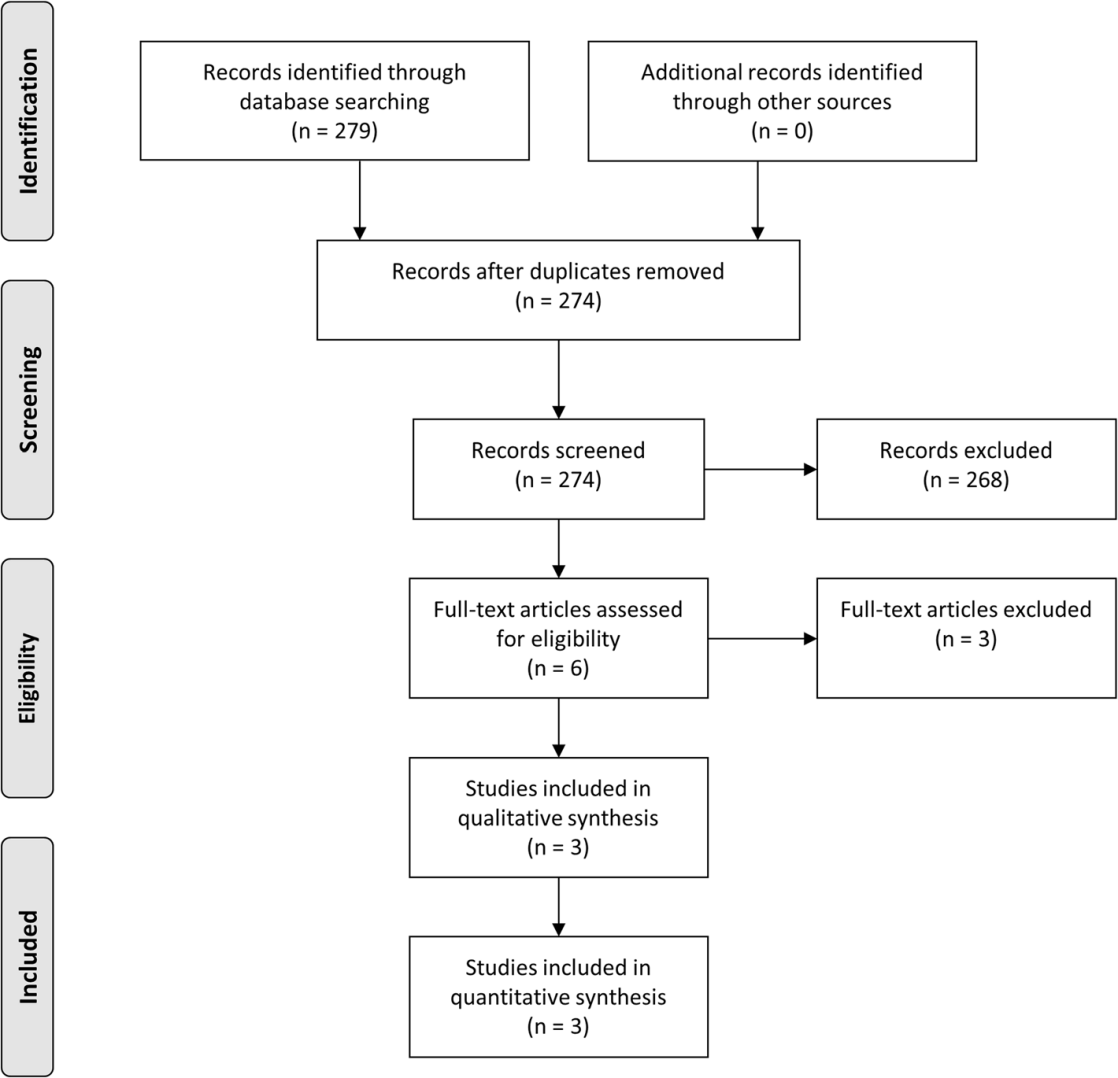
Flow chart of the comprehensive literature search according to PRISMA Guidelines

The comprehensive computer literature searched from PubMed, Cochrane Library and MEDLINE databases revealed 279 articles. Results were matched and 5 duplicated studies were excluded. Reviewing titles and abstracts, 268 records were excluded because did not match the main topic. Three articles were excluded since they did not respect the inclusion criteria. Three articles including 605 patients were selected and were eligible for the meta-analysis (Fig. 1); no additional studies were found screening the references of these articles.

### Study characteristics

**Fig. 2:**
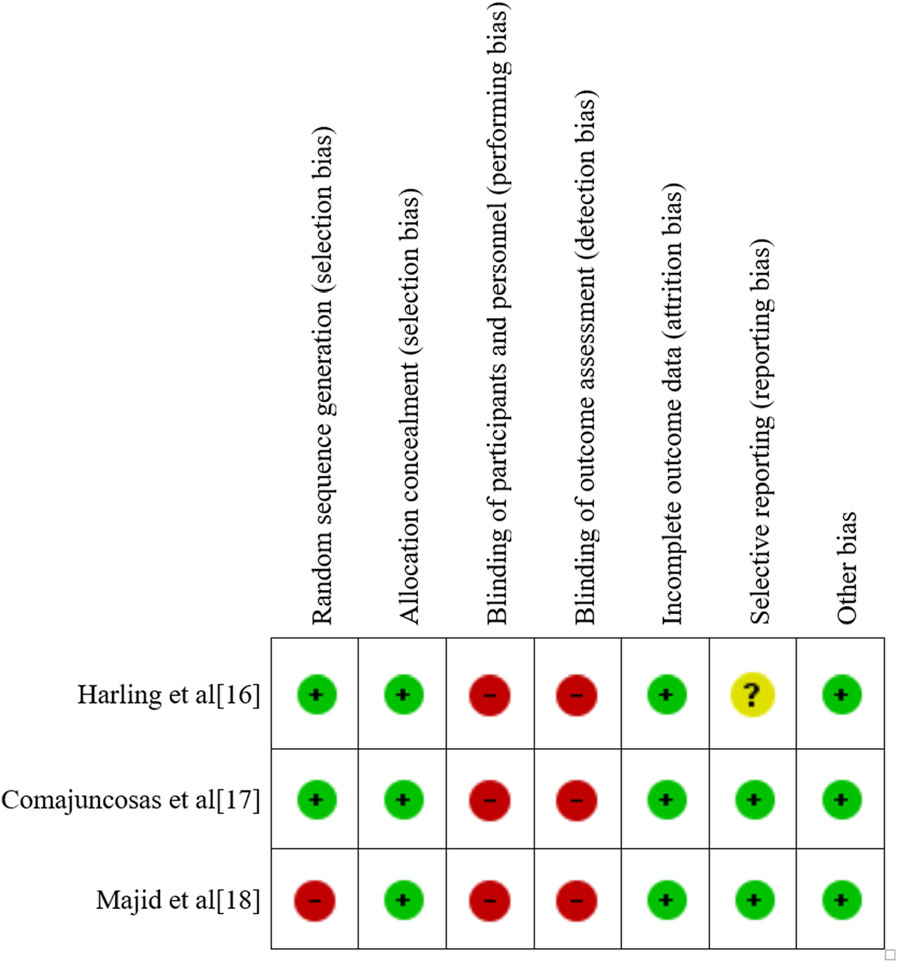
Risk of bias assessment of included studies

The eligible studies were 2 randomized trials [16, 17] and a multicenter prospective study [18]. Harling et al. [16] compared groups receiving a dose of cefuroxime (750 mg i.v.) or having the gallbladder removed with a retrieval bag. SSI was defined as pus discharging from the wound with signs of inflammation. Comajuncosas et al. [17] randomly assigned patients to have the gallbladder extracted with a retrieval bag or not. SSI was a defined as a positive culture and/or a signs of infection. Majid et al. [18] compared the use of a retrieval bag to extract the gallbladder with no use of the retrieval bag in a population receiving preoperatively a dose of 1.2 g Co-Amoxiclav. SSI was defined as an infection requiring antibiotics with or without drainage. The follow up was 30 days for all the studies. The risk of bias among included studies is reported in Fig. 2. According to the Study Quality Assessment of Controlled Intervention Studies NHLBI [11], the study of Harling et al. [16] and Comajuncosas et al. [17] were categorized “good” and the study of Majid et al. [18] was categorized “fair”. According to the GRADE approach, due to sparse data (lack of directness rated down by 1) and limitations in studies quality (lack of blinding rated down by 1), the overall quality of evidence of this meta-analysis was judged “low”.

### Statistical results

**Fig. 3:**
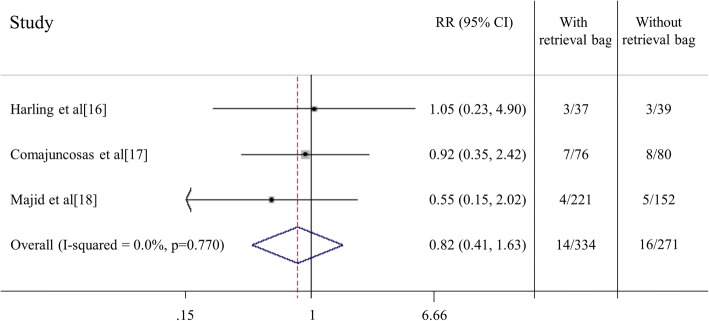
Forest plot of the statistical analysis described the relative risk (RR), the 95% confidence interval (CI) and the subtotal I-squared for all the studies included. Harling et al. [16], Comajuncosas et al. [17] and Majid et al. [18] studies

**Fig. 4:**
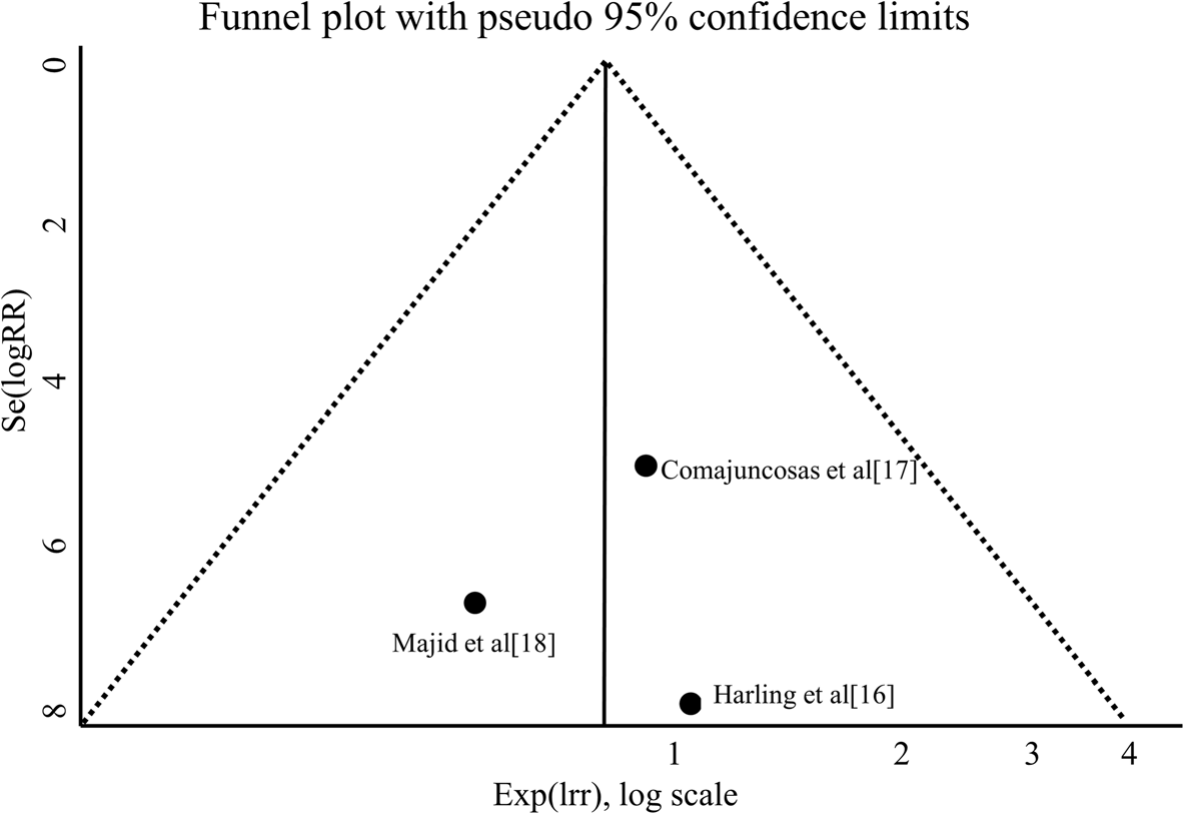
The funnel-plot for publication bias assessment showed no noteworthy pattern. Harling et al. [16], Comajuncosas et al. [17] and Majid et al. [18] studies

Wound infections were documented in 14 of 334 (4,2%) patients operated using a retrieval bag versus 16 of 271 (5,9%) patients operated without the use of a retrieval bag (Additional file 1). The statistical analysis revealed a RR of 0.82 (0.41–1.63 95% CI) indicating no statistically significant reduction in wound infection through the routine use of a retrieval bag to extract the gallbladder from the abdominal cavity (Fig. 3). Concerning sensitivity analysis the estimated pooled risk ratio ranged from 0.72 to 0.96 omitting Comajuncosas et al. [17] or Majid et al. [18] studies, respectively, both not statistically significant. Harbord test did not reveal the occurrence of small-study effect (*p* = 0.892) and the funnel-plot showed no noteworthy pattern (Fig. 4).

## Discussion

SSIs are responsible for increased length of hospitalization and health care costs. Diabetes, malnutrition, male gender, chronic anemia, obesity, drug abuse, smoking-related diseases and previous *Staphylococcus aureus* infection were reported in several studies as patient-related risk factors for SSIs after cholecystectomy [19].

In order to avoid surgical site contamination from bile and stone spillage, surgeons pay attention not to open the gallbladder during its dissection from the liver bed and retrieval from the abdominal cavity. Bile in the gallbladder or bile ducts in the absence of gallstones or biliary tract disease is normally sterile. In the presence of gallstones, the prevalence of bacteria increases: the percentage of positive gallbladder bile cultures among patients with symptomatic gallstones and chronic cholecystitis ranges from 11 to 30%. Positive bile cultures are significantly more common in elderly (> 60 years) patients with symptomatic gallstones than in younger patients (45% versus 16%) [20].

From the early days of laparoscopic surgery, many manufacturers have developed different types of retrieval bags, whose use has become popular among minimal-invasive surgeons in laparoscopic appendectomy, cholecystectomy, bowel resection and annexectomy.

According to the “Guidelines for the Clinical Application of Laparoscopic Biliary Tract Surgery” of the Society of American Gastrointestinal and Endoscopic Surgeons (SAGES), the use of an endoscopic bag is left at the discretion of the operating surgeon [2]. In order to evaluate the popularity of retrieval bags in elective laparoscopic cholecystectomy, we sent a questionnaire via email to 150 consultant general surgeons working in 5 different countries (Switzerland, United Kingdom, Germany, Italy and Austria), asking whether and why they would routinely use an extraction bag during this procedure. We received an answer from 61 surgeons (40.7%). With one exception, all those interviewed (98%) confirmed the routine use of this device. Twenty-five (41%) surgeons justified it by the opinion that it contributed to prevent a wound infection at the trocar site. The other surgeons answered with “comfort” or “no reason”. Clearly, it should be taken into account that the retrieval bag gives the possibility to remove spilt stones and to aid extraction in patients affected by morbid obesity and when the gallbladder has been opened during the dissection [3].

The incidental gallbladder cancer is considered a rare eventuality happening in 0.2–3.3% of elective cholecystectomies [21]. In fact, 50% of cases are identified preoperatively and 29% intraoperatively. Only 21% of cases are recognized at the definitive histologic examination [22]. In such cases, the routine use of a retrieval bag to prevent post-site metastasis was claimed to be mandatory [3], even though several factors should be considered. In addition to the rarity of the disease, it is described that almost 90% of patients do not develop a port metastasis. In case of port-metastasis, this is localized in 53% of cases at the site of the extraction trocar, being the risk in non-extraction trocars 47% [22].According to some authors, a port-site metastasis reflects more likely the tumor wide-spreading and the aggressive biology rather than a direct contact of the gallbladder with abdominal wall [22]. Moreover, data of the central register of “incidental gallbladder carcinoma” of the German Society of Surgery suggested that the usage of retrieval bags was not associated with a decreased risk of seeding if gallbladder perforation did not occur intraoperatively [3]. Finally, in case port metastasis occur, the port-sites excision could be a valuable therapeutic option, as this surgical step is nowadays still a matter of debate [22, 23]. To date, these elements made the prophylactic use of a retrieval bag to avoid neoplastic cells seeding in all elective cholecystectomies widely debatable.

In case of acute cholecystitis, many authors recommend the extraction of the gallbladder in a retrieval bag as port site infections are frequently associated with spillage of infected bile, stones or pus [4–6]. Even if the use of a retrieval bag in the above-mentioned situations seems justified or reasonable, there is no strong evidence to support the use of a retrieval bag in elective laparoscopic cholecystectomy. All wound infections in the study of Harling et al. [16] were associated with skin commensals. In the study of Comajuncosas et al. [17], in all cases, except one (*E. coli*), organisms isolated from the wound sites of those patients that developed postoperative infections were skin commensals (Corynebacterineae, coagulase-negative Staphylococcus spp., Streptococcus pyogenes). In previous studies, similar results were obtained [24–30]. The absence of correlation between typical (gram negative) bile and wound infection organisms suggest that most port site infections do not depend on a direct contact of the gallbladder with the wound.

In our meta-analysis a SSI was documented in 14 of 334 (4,2%) patients operated using a retrieval bag versus 16 of 271 (5,9%) patients operated without the use of a retrieval bag. The statistical analysis revealed a RR of 0.82 (0.41–1.63 95% CI) and no statistically significant reduction in SSI when the extraction of the gallbladder from the abdominal cavity was performed with a retrieval bag.

The latter, in addition, is not risk-free. In the largest study of our meta-analysis [18], an enlargement of the port site incision was required in 9,7% (36/373) of patients. At 1 year follow-up, there was no recorded cases of port site hernia in the group without the use of a retrieval bag and two (0,9%) cases of port site hernia in the retrieval bag group. In addition, there is an anecdotal risk of abdominal organs damage during bag insertion and retrieval [7–9].

Retrieval bags are not cheap, ranging from € 25 to € 120, and their use must be questioned in a time of rising economic pressure on the health care providers. Interestingly, there are plenty of reports in the medical literature about “cost-effective, self-made” specimen extraction bags [31–34].

This study has several limitations. A small number of trials were eligible for the meta-analysis, resulting in a low number (605 altogether) of patients. The study of Majid et al. [18] albeit prospective, is non-randomized. Another limitation is the mean prevalence of SSIs in the included studies. It is stated that an acceptable SSIs rate ranges between 1.6 and 3.2% [35, 36], defined according to Centers for Disease Control [37] as purulent discharge from the surgical site, with or without positive culture or signs of inflammation. However, in our analysis it was 7.9% in Harling et al. [16], 9.6% in Comajuncosas et al. [17] and 2.4% in Majid et al. [18] studies. The high incidence of SSI could be explained by different definitions adopted. Nevertheless, regardless the absolute number of infections, the primary endpoint of our study was the evaluation of postoperative SSI rate, which resulted equal between groups in all studies. Considering the lack of significant difference related between groups, the cause of infection is improbable to be related to the direct contact of the bile and gallbladder with the wound.

Moreover, the included studies have different sample sizes, being the majority of patients in the study of Majid et al. [18]. In order to evaluate this possible bias, we assessed the sensitivity analysis (ranging from 0.72 to 0.96) and it was not statistically significant. Harbord test did not reveal the occurrence of small-study effect and the funnel-plot showed no noteworthy pattern, both indicating this bias unlikely.

Another limitation is the heterogeneity in the antibiotic prophylaxis regimens. In the study of Harling et al. [16], the patients were randomized to receive a single dose of Cefuroxime (750 mg, i.v.) or to have the gallbladder removed from the abdomen with a retrieval bag. Comajuncosas et al. [17] used no antimicrobial prophylaxis, Majid et al. [18] gave a single dose of 1.2 g Co-Amoxiclav at the time of induction (1,5 g Cefuroxime in case of penicillin allergy). However, the use of retrieval bag did not change the rate of SSI, nor in presence or absence of antimicrobial prophylaxis, neither compared with antibiotics administration.

We applied the GRADE approach [12] in order to evaluate the quality and, taking into account limitations mentioned above, the quality of evidence of our paper ranked from “moderate” to “low”.

## Conclusions

The results of this review highlight the paucity of well-designed large studies and despite limitations related to the low level of evidence, our meta-analysis showed no significant benefit of retrieval bags in reducing the infection rate after elective laparoscopic cholecystectomy. In absence of acute cholecystitis, accidental intraoperative gallbladder perforation or suspected carcinoma their use, to date, may not be mandatory, so that, further studies focusing on complex cases are needed.

## Additional file

Additional file 1: Dataset used for the statistical analysis. (XLSX 8 kb)

## Abbreviations

CI: Confidence interval
GRADE: Grading of Recommendations, Assessment, Development and Evaluations
PRISMA: Preferred Reporting Items for Systematic Reviews and Meta-Analyses
SSI: Surgical site infection
